# The History, Diagnosis, and Treatment of Typhoid and Typhus Fever; with an Essay on the Diagnosis of Bilious Remittent and of Yellow Fever

**Published:** 1842-10-29

**Authors:** 


### BIBLIOGRAPHICAL NOTICE.

*The History, Diagnosis, and Treatment of Typhoid and of Typhus Fever; with an Essay on the Diagnosis of Bilious Remittent and of Yellow Fever.* By Elisha Bartlett, M. D., Professor of the Theory and Practice of Medicine in the Transylvania University. Philadelphia: Lea & Blanchard. 1842. 8vo. pp. 393.

This is a very handsome volume, which has occupied a portion of the leisure of Professor Bartlett during the interval between the winter lectures at the Transylvania University. The author has given us a work much wanted. We will give his own reasons for writing it.

"I have written this book, because I thought, that I saw a want in medical literature, which it might supply. Our science, so far as the great subject of idiopathic fevers is concerned, is passing through a transition period; and many authorities, that were received as standard and classical, only a few years ago, are fast becoming obsolete; at least for American readers. This is particularly true of the leading English treatises on Fever. Neither the works of Fordyce, Armstrong, Southwood Smith, nor Tweedie; nor the elaborate articles on Fever, in the Medical Cyclopedias, Libraries and Dictionaries, can henceforth be regarded as sufficient, or even safe guides for American practitioners; and the remark is applicable to them, not because they are not works of great excellence and value; but for other reasons, which will be abundantly obvious in the course of the following pages. I may simply say, here, that their authors describe, principally, a fever, which is rarely met with in this country; and that they do not represent the actual state of our knowledge upon this subject. It must be regarded as especially unfortunate, that, until within a few years, the greater part of our information, relating to continued fever, has been derived from writers, who have treated, mostly, and under the same name as that generally used by ourselves, of a disease, or form of disease, differing, in many important respects, from that which is most common with us; and, that, in this way, so great a degree of confusion has been introduced into our notions of fever."

The author begins his hisiory of the two diseases by that of Typhoid fever. He admits, as every one must do who has given much attention to the subject, that it is a well characterised disease, equally so in America as in Europe, and one that prevails extensively in many parts of the United States. Referring to individual cases of the disease given in American publications, to show that it is really identical with the fever described by Dr. Louis, he next proceeds to the analysis of the symptoms, pathology and treatment of the fever. The sources from which he has drawn most largely are, the publications of Drs. Louis and Chomel, and the memoirs of Drs. Jackson and Hale, of Boston. As the disease prevails largely in most parts of New England, Dr. Bartlett, during a residence of nine years at Lowell, saw many cases of the disease amongst the factory girls of that place, most of whom are of the age at which the fever is most prevalent. His own experience includes a severe epidemic, besides the sporadic cases which are constantly occurring. A publication of older date than those above mentioned, that of Dr. Smith, furnishes a large number of interesting facts, which are judiciously compared and incorporated with those of more recent date.

The very nature of the memoir on Typhoid admits almost nothing which can be called novel; it is just what it is designed to be, a digest of the books published on the subject, which gives within a moderate compass the matter scattered through many volumes.

The pathology and symptomatology of typhoid fever are fully settled, and Dr. Bartlett therefore was able to give us the actual results of the observations of many authors: but as regards the treatment of the disorder, it is not yet settled what is the best possible treatment in all cases. So far as the chief indications are concerned, there is very little difference of opinion, and by trusting to the remedies suggested naturally by the varying symptoms, we cannot be far from right. Topical and even general bleeding, when there is much excitement, laxatives and mild alteratives towards the close of the disease, are the best remedies as *a general* rule, but the modifications are numberless. Dr. Bartlett very properly remarks that, "for the present, our management of the disease must be eclectic and rational, not exclusive and specific."

A second memoir is on the subject of Typhus fever. Dr. Bartlett admits the distinction between the two great forms of continued fever, and could find no evidence of an unequivocal kind to show that the diseases were really identical. The only apparent exception is that of the fever described by Dr. Landouzy, as occurring in the prison at Rheims. This was a typhoid fever, with some of the symptoms of typhus; the latter, perhaps, produced by the unfavourable circumstances in which the patients were placed. Such combinations are by no means rare in other diseases: typhoid fever during the present year was evidently at times connected with remittent, and the paroxysmal character was well marked; pneumonia, as every one knows, is modified to a very great degree, and the same remarks may be applied to other disorders. The great landmarks, those which constitute the signs of identity, remain, however, and the disease is characterised, though its symptoms are slightly modified or tinged by those of another disorder.

The materials upon which Dr. Bartlett has depended for his memoir on Typhus, consist chiefly of the excellent memoirs of the Irish physicians, Cheyne, Graves and others: the memoirs of Smith, Hudson, Davidson and other writers; a memoir by Dr. Gerhard in the American Journal; one by Dr. Shattuck in the Medical Examiner, &c. The only work which we regret not to see mentioned, is that of Hildebrand, which would have shown very clearly that the camp fever of the European armies in 1813 was really typhus.

Dr. Bartlet admits fully that the symptoms, the age of the patients, the mode of transmission, and the pathological anatomy of typhus, differ much from those of typhoid fever, and therefore considers the disease as distinct.

The work terminates with two short memoirs on Remittent and Yellow Fever, chiefly with reference to their diagnostic characters.

We have not attempted to give an analysis of the work, because it is itself an analysis, and a very condensed one of the writings of many authors. The style is clear and terse, and what we value still higher, the work is written in a spirit free from prejudice, and is restricted within the limits which the facts warrant.

We trust that it will be appreciated as it ought to be, and meet with an extended circulation, especially in those parts of the country in which the fever is prevalent.

